# Therapeutic Patient Education after Anterior Cruciate Ligament Reconstruction: Evaluation of the Knowledge and Certitudes with a Self-Report Questionnaire

**DOI:** 10.3390/healthcare10050934

**Published:** 2022-05-18

**Authors:** Alban Fouasson-Chailloux, Vincent Crenn, Bastien Louguet, Jérôme Grondin, Pierre Menu, Marc Dauty

**Affiliations:** 1 Service de Médecine Physique et Réadaptation Locomotrice, Nantes University, University Hospital of Nantes, Hôpital St. Jacques, 85 Rue Saint Jacques, 44093 Nantes, France; jerome.grondin@chu-nantes.fr (J.G.); pierre.menu@chu-nantes.fr (P.M.); marc.dauty@chu-nantes.fr (M.D.); 2 Service de Médecine du Sport, Nantes University, University Hospital of Nantes, Hôpital St. Jacques, 85 Rue Saint Jacques, 44093 Nantes, France; bastien.louguet@chu-nantes.fr; 3 INSERM UMR 1229, RMeS, Regenerative Medicine and Skeleton, Nantes University, 44000 Nantes, France; 4 IRMS, Institut Régional de Médecine du Sport, Hôpital St. Jacques, 85 Rue Saint Jacques, 44093 Nantes, France; 5 Clinique Chirurgicale Orthopédique et Traumatologique, Nantes University, Hôtel-Dieu, 44000 Nantes, France; vincent.crenn@chu-nantes.fr

**Keywords:** knee, ACL injury, sport, hamstring, strength, laxity

## Abstract

Therapeutic education aims to help patients acquire skills and knowledge, and to improve psychosocial aspects to manage chronic disease. After anterior cruciate ligament reconstruction (ACLR), only 35 to 60% of the patients are able to go back to their previous sport. Return to sport depends on the motivation of the patient. No therapeutic education has already been proposed. We aimed to evaluate the effect of therapeutic education sessions on knowledge improvement during inpatient rehabilitation after ACLR, compared to patients operated with the same surgical technic, but who had no therapeutic education because of outpatient rehabilitation. Sessions were performed by a multidisciplinary team. The evaluation of the knowledge was performed with a true or false 12-items self-report questionnaire. Fifty-four patients were studied and compared to 54 patients with no therapeutic education. The educated and the non-educated groups were comparable. The number of correct answers increased from 73% before therapeutic education to 95% at the end of the hospitalization (*p* < 0.001). This improvement persisted over time with 91.5% of correct answers at four months (*p* = 0.94). The non-educated group had 70% of correct answers. This was significantly lower than the results obtained from the educated group at four months (*p* < 0.001). It was comparable to the result obtained before therapeutic education (*p* = 0.91). Therapeutic patient education performed during hospitalization for rehabilitation enables patients to have a better knowledge of the stages from rehabilitation to return to sport and the risks of complication after ACLR.

## 1. Introduction

Therapeutic education aims to help patients acquire skills and knowledge in order to manage chronic disease in their daily life [1]. It concerns activities designed and organized to enable patients to better understand their disease and their treatments, and to take responsibility of their own medical management in order to maintain and improve their health status. Therapeutic patient education seeks to highlight objectively and subjectively the modifications of patients according to three aspects: pedagogical, psychosocial and bio-clinical [2,3]. Its effectiveness has been demonstrated in chronic diseases such as diabetes mellitus, asthma, high blood pressure, obesity or chronic obstructive pulmonary disease [4,5,6].

The annual incidence of the anterior cruciate ligament reconstruction (ACLR) is from 30 to 45 per 100,000 [7,8]. Twelve months after surgery, only 35 to 60% of the patients are able to go back to their previous sport at the same level [9,10]. Return to sport depends on the motivation of the patients to practice physical activities and their motivation during rehabilitation [11,12,13]. This is the reason why psychological questionnaires have been developed to evaluate patients’ confidence in their own operated knee [14,15], but to our knowledge no therapeutic education has already been proposed. Therapeutic education could be interesting to help patients go back to their previous sports while preserving their operated knee. After ACLR, therapeutic patient education aims are:

The improvement of the bio-clinical aspect concerning the operated knee, such as pain, stiffness, infection and anterior cruciate ligament (ACL) rupture recidivism [16].

The improvement of the psychosocial aspect, that is to say, fighting against the fear of the rehabilitation, the fear of physical activities and the fear of returning to sport; and performing self-exercises of rehabilitation and physical self-trainings [17,18].

The improvement of the knowledge and the ability to self-assess [19]. The knowledge is declarative when concerning time and criteria to return to sport, and the clinical warning signs. The knowledge is procedural when it creates skills for the behavior to have in case of complications or for realization of self-exercises. The trust that the patients place in their own knowledge is essential for the return to sport at the same level [20].

We aimed to evaluate knowledge and certitudes about medical issues, rehabilitation and, return to daily-life and sport, for patients who had undergone an ACLR with hamstring tendon autograft during hospitalization for rehabilitation. The evaluations were made with self-report questionnaires to measure the effect of therapeutic education sessions on knowledge and certitudes improvement. Four months after surgery, the patients were compared with other sports patients operated with the same surgical technic, but no therapeutic education because of an ambulatory rehabilitation.

## 2. Materials and Methods

### 2.1. Participants

The study took place in the rehabilitation department of the University Hospital of Nantes, France. In our hospital, patients can be addressed by their surgeons either right after the surgery to perform their early rehabilitation in our center, or addressed only at four months after surgery for knee recovery evaluation. We assessed sports patients over 18 years old, who had rehabilitation after ACLR with hamstring autograft during the year, from January to December 2018. We compared patients who had their initial rehabilitation in our center to patients only assessed at four months. Indeed, the patients hospitalized for rehabilitation performed as a routine a self-report questionnaire, before therapeutic education, at the end of the rehabilitation program, and four months after surgery during a consultation of follow-up to assess strength recovery of their operated knee [21,22]. The patients who had ambulatory rehabilitation performed the same questionnaire four months after surgery, without therapeutic education. Six different surgeons had operated on the patients and gave them the choice between the rehabilitation center or ambulatory management with a physiotherapist. Neither the surgeons nor the patients knew that a therapeutic education would be performed in case of rehabilitation at hospital.

Age, gender and socio-professional category were reported according to the French job classification—Professions and socio-professional categories (PSC 2003)—of the Institut National de la Statistique et des Etudes Economiques (INSEE) nomenclature [23]. The professional status of the patients gave information about their learning abilities.

All the patients received an accelerated rehabilitation program of their operated knee [24]. Four months after surgery, all the patients performed a self-report questionnaire during a consultation to assess muscle recovery.

To be included, all the patients should have had an ACLR with hamstring tendon autograft technic. In the therapeutic education group, patients had to perform the educative program during inpatient rehabilitation and to perform the consultation of follow-up at four months. In the other group, patients had only to perform the clinical evaluation at four months.

The criteria of exclusion were: (1) patients operated on with other surgical techniques, especially with patellar tendon grafts because of inadequate questions, (2) patients who had an ACL revision surgery or who had previously had an operation on a knee, (3) professional and high level athletes because of the need to return quickly to sport.

### 2.2. Therapeutic Education Sessions

Therapeutic education sessions were performed as routine by a multidisciplinary team composed of three physiotherapists and a specialist in adapted physical activity, managed by a physician specializing in physical medicine and rehabilitation. The successive steps of the session program were realized as follows:

(1) The educational diagnosis was performed at the beginning of the hospitalization, after patient’s consent, during an individual interview with one of the members of the therapeutic education team. This diagnosis allowed to collect information about the patients (personality, demands, short- and long-term projects) in addition to psycho-social information. An information booklet on surgical procedures, rehabilitation and therapeutic education was delivered at the end of the interview. Demographic, anthropometric, surgical, sports and professional characteristics were also reported.
(2) The therapeutic education contract or therapeutic alliance was performed from the synthesis of the educational diagnosis in order to define the individual objectives of the patients and to work on their skills during rehabilitation sessions.
(3) Two to four patients were gathered during the sessions, according to their individual objectives. The improvement of the knowledge concerning the stages from the surgery to the return to competition sport, were performed with pictures that the patients had to classify in chronological order. The improvement of their skills concerning the security of their operated knee was performed thanks to an understanding of the clinical signs of knee complication and the behaviors to follow. Overall improvement of the knowledge was achieved using green, red or white answers to oral proposals (green = true; red = false; white = do not know).

The evaluation of the knowledge was performed with a true or false self-report questionnaire associated with the degree of certitude of the answer (50–60–80–100%). This questionnaire was filled out at the beginning and at the end of the hospitalization for rehabilitation [25]. This questionnaire included 12 questions (Table 1).

### 2.3. Self-Report Questionnaire Conception

The 12 questions were designed thanks to a literature review, professional recommendations and the most frequently questions asked by the patients. The patients often asked questions 5, 8, 10 and 12. Questions 5, 8, 10 and 12 were declarative and assessed general medical and athletic knowledge [16,26,27]. Questions 1, 3, 6 and 9 were procedural and specific to rehabilitation and return to sport [17,28,29,30]. Questions 2, 4, 7 and 11 were procedural and logical [31,32,33]. 

This self-report questionnaire was initially validated with a population of recreational athletes (*n* = 30) without knee injury, according to methods described in the literature [14]. The test–retest reproducibility was good with an intra-class correlation coefficient of 0.794 (0.555–0.899; *p* < 0.0001) and a feasibility requiring an average realization time of 145 ± 28 s. The internal consistency was good with a strong correlation between the 12 questions (alpha coefficient of Cronbach at 0.826). The discriminative validity was fair with a significant difference between the correct answers of the population of athletes without knee injury and the educated patients operated on the knee (*p* < 0.0001). 

### 2.4. Statistical Analysis

The results of the self-report questionnaire were presented in the form of a spectral distribution with the number of correct and incorrect answers, before therapeutic education, three weeks and four months after surgery. The results were compared with an ANOVA test and post-hoc test of Bonferroni. The knowledge was considered perfect when the degree of certitude was 100%. The knowledge was partial in case of degree of certitude of 60 and 80%. Ignorance was highlighted in the case of right or wrong answers with a degree of certitude of 50%. The misconceptions were considered in the case of incorrect answers with a degree of certitude of at least 60%. They were considered serious or even dangerous in the case of degree of certitude of 100% [25]. The two groups were compared four months after surgery with a Student’s t-test for the quantitative parameters and a χ^2^—test for the qualitative parameters. Statistical analysis was performed with SPSS 23.0^®^ (IBM, Armonk, NY, USA). The results were considered significant at the 5% critical level (*p* < 0.05).

## 3. Results

Sixty recreational athletes were included and received education during their hospitalization after ACLR with hamstring autograft. Six were lost to follow-up because they did not come to the clinical evaluation four months after surgery. So, 54 patients were finally studied at four months and compared to 54 consecutive patients with no therapeutic education who, during the same period, performed the self-report questionnaire during the follow-up consultation, in order to have the same number of patients in both groups. The educated and the non-educated groups were comparable concerning demographic, anthropometric, sports and professional characteristics (Table 2).

The knowledge improved significantly in the educated group, the number of correct answers increased from 73% before therapeutic education to 95% at the end of the hospitalization (*p* < 0.001). This improvement persisted over time with 91.5% of correct answers four months after surgery (*p* = 0.94). In comparison, the non-educated group had 70% correct answers. This result was significantly lower than the results obtained from the educated group at four months after surgery (*p* < 0.001). It was comparable to the result obtained before therapeutic education (*p* = 0.91).

The certitudes also improved significantly because the perfect knowledge (correct answers with degree of certitude of 100%) increased from 30 to 84.5% after therapeutic education (*p* < 0.001). The certitudes were confirmed at four months after surgery (79.5%). The non-educated group had perfect knowledge in only 33.5% of the cases, which was similar to the other group before therapeutic education (*p* = 0.87). Yet, the serious misconceptions (incorrect answers with degree of certitude of 100%) remained stable with 5.5% before therapeutic education and 3% at three weeks after education and 3.8% at four months. The non-educated group had 9.4% of serious misconceptions, that was not different from the group before education (*p* = 0.21).

**Figure 1:**
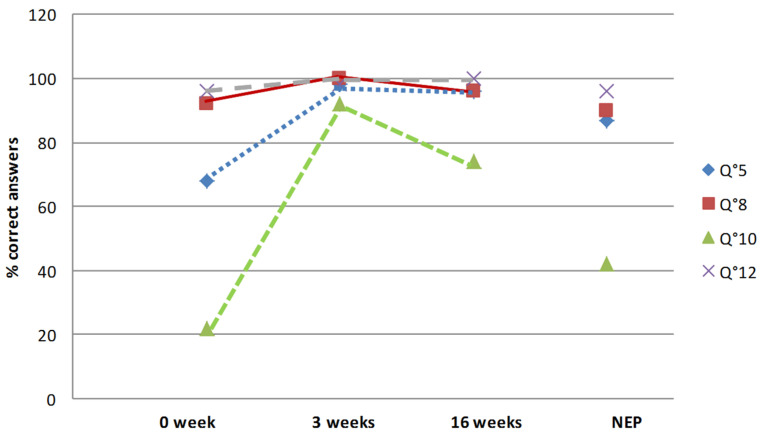
Evolution of the answers to the declarative questions about medical and sports knowledge (Q5, Q8, Q10 and Q12) during the therapeutic education period for the patients who had inpatient rehabilitation, and answers at four months for the outpatient rehabilitation group. Abbreviation: NEP: non-educated population (outpatient rehabilitation group).

The rate of right answers depended on the type of questions. The answers to the declarative questions evaluating medical and sportive knowledge were usually correct before therapeutic education (questions 5, 8, 10 and 12). Questions 8 and 12 had, respectively, 92 and 96% of right answers with a high degree of certitude (Figure 1). Indeed, the patients already knew that return to playing football four months after surgery was unusual (question 8) and that there was always a risk of ACL reconstruction failure (question 12). The answers to question 5, which concerned protection of the scar from the sun, were correct (68 and 87% for the educated group and the non-educated one, respectively). Question 10 (saying that swimming was as efficient as cycling for strengthening the muscles of the thigh) was the most difficult question of the self-report questionnaire because there was only 22% of correct answers before education and 42% in the non-educated group (*p* = 0.03).

**Figure 2:**
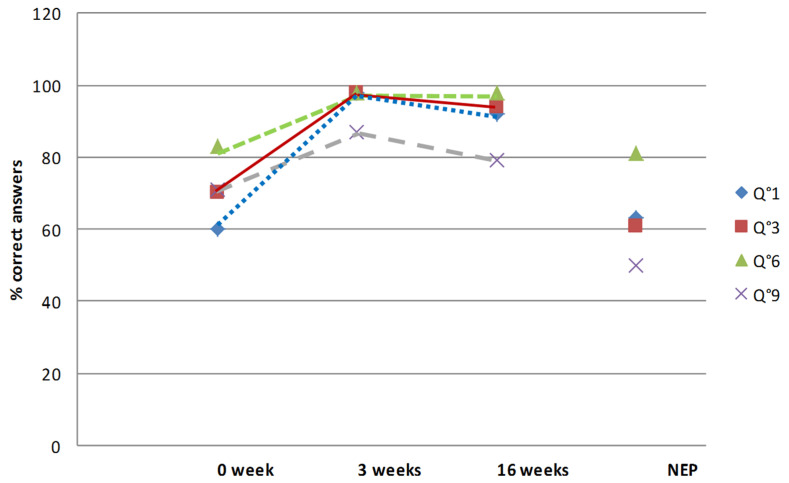
Evolution of the answers to the procedural questions relative to rehabilitation and return to sport (Q1, Q3, Q6 and Q9) during the therapeutic education period for the patients who had inpatient rehabilitation, and answers at four months for the outpatient rehabilitation group. Abbreviation: NEP: non-educated population (outpatient rehabilitation group).

The procedural questions (1, 3, 6 and 9) were specific to rehabilitation and return to sport. The answers to question 6 were immediately correct (83% in the educated group and 81% in the non-educated one) (Figure 2). Question 6 was about the priority of knee range of motion recovery. Questions 1, 3 and 9 were the most difficult ones. Waiting for the knee strength assessment at four months to decide to start jogging (question 1) was clearly non-effective (60% of correct answers in the educated group and 63% in the non-educated one). The results were similar concerning the return to collective sports six months after surgery (question 3), with 70% and 61% of correct answers in the respective groups. Waiting for the recovery of the knee range of motion before starting cycling (question 9) was a difficult question with only 71% and 50% of correct answers in the respective groups.

**Figure 3:**
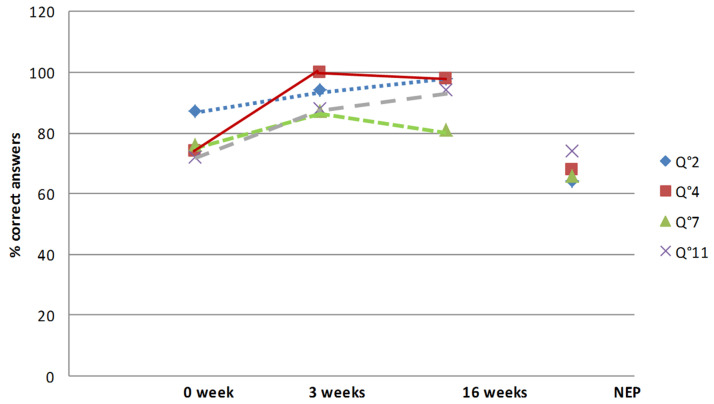
Evolution of the answers to procedural and logical questions (Q2, Q4, Q7 and Q11) during the therapeutic education period for the patients who had inpatient rehabilitation, and answers at four months for the outpatient rehabilitation group. Abbreviation: NEP: non-educated population (outpatient rehabilitation group).

The answers to the procedural and logical questions (2, 4, 7 and 11) were correct in 64 to 87% in the two groups (Figure 3). Stopping the cycling program in case of knee swelling (question 2) was partly acquired (87% of initial correct answers in the educated group and 64% in the non-educated one). The initial answer to the question concerning the protection of the hamstrings after rehabilitation (question 4) was correct in 74% of cases in the educated group and 68% the non-educated one. The impossibility of driving in case of walking sticks (question 7) was initially known for 76% of the patients of the educated group and 66% for the other group. Seventy-two and 74% of the patients, in the respective groups, already knew that return to sport should be authorized by the sports physician (question 11).

After therapeutic education during the hospital stay, the rate of correct answers increased for all the questions, from 87 to 100% of the correct answers. At four months after surgery, in the educated group, the rate of correct answers remained high (from 74 to 100%). Questions 9 and 10 remained the most difficult questions even after education. Yet, concerning question 10 about swimming, progress was considerable because the rate of correct answers increased from 22 to 74. On the other hand, the answers to question 9 dealing with knee range of motion remained low despite therapeutic education (from 71 to 79%). 

In the educated group, the certitudes significantly increased for all the questions. Before education, perfect knowledge was from 3.7% (question 10) to 62.9% (question 12). After education, perfect knowledge increased significantly and was between 68.5% (question 9) and 94.4% (question 5). Four months after surgery, perfect knowledge had persisted, from 64.8% (questions 9 and 10) to 94.4 (question 12). Yet, serious misconceptions remained stable. Indeed, before education, the higher rate of misconceptions was 27.7% for question 10; after education the higher rate was for question 7 (12.2%) and at four months for question 10 (14.8%). The rate of serious misconceptions was higher after education than before for question 11, but it remained low (1.8% vs. 5.5%).

## 4. Discussion

Therapeutic education sessions after ACLR significantly improved the knowledge and certitudes of the patients. These results have persisted with time for the patients who had a therapeutic education, and the results were significantly higher than those of a non-educated group four months after surgery. It demonstrates the value of the educational approach in this context. The evaluation according to the degree of certitude was innovative for a surgical pathology, considered nonchronic compared to other diseases such as diabetes mellitus, chronic bronchitis, cardiovascular diseases or rheumatologic diseases [4,6]. Yet, therapeutic education seemed justified because of potentially serious complications after surgery and the delay to return to sport (which is the goal of the surgery) [10,12,34]. The complications usually concern one-third of the patients [22]. The risk of infection, pain or knee stiffness exists especially during the first year after surgery, and the risk of ACL graft rupture persists the whole life of the patient, particularly during sport practice [35,36]. Yet, our results are difficult to compare with other studies as our approach seems to be relatively new in the context of ACLR.

In detail, the questions about the specificities of rehabilitation (questions 2, 4 and 6) showed a weakness of knowledge, which is worrying, because all the patients have physiotherapy care after serious knee sprain and usually before surgery. This could reflect a lack of explanations from the therapist when performing the care. The misconceptions observed for question 7 about the possibility of driving with walking sticks, pose the problem of the absence of information given by the prescriber and the dangerous behavior of the patients. This is all the more serious as these misconceptions have persisted even after therapeutic education.

Some questions concerning return to sport (questions 1, 3 and 8) may have been redundant because they analyzed the same field of knowledge. Yet, it is a frequent issue because patients mostly undergo surgery in order to go back to an unsafe sport for the operated knee [9,17]. So, any misconception appears dangerous. It could be secondary to incorrect information provided by the media and the sport staff, who usually generalize the exceptional follow-up of high-level athletes, which is far different from those of recreational athletes.

Question 10 was the most difficult one even if it was about the interest of cycling or swimming to improve muscle strength. This question had the higher rate of serious misconceptions. This question involved very specialized knowledge with errors resistant to therapeutic education. Fortunately, it has no impact on the endangerment of patients. In order to improve the answers to this question, it would be necessary to assess patients’ comprehension of the questions because these two types of training are usually allowed before the four months after surgery [25]. Conversely, question 12 about the rupture of the graft was easier with a high level of good responses in both groups. This is reassuring because this question is essential to avoid risky behaviors. Indeed, this information may have been provided by the surgeon.

The study has some limitations. Firstly, the therapeutic patient education sessions were performed during hospitalization but it is not possible to know if the hospitalization helped the processes of learning. The comparison with a non-educated group four months after surgery, only allows to confirm that the level of knowledge and certitudes of this group was comparable with those of the educated group before hospitalization and education. It is surprising that the follow-up with the surgeon and the management with a physiotherapist in case of external care did not allow a better knowledge of the pathology. Secondly, our study is limited by its design. Indeed, the groups were not randomized due to the type of recruitment, which might have an impact on the comparability of the patients, but it was difficult in our case as the control group was addressed only four months after surgery by surgeons. Thirdly, concerning the self-report questionnaire, it seems specific to our population. Indeed, some correct answers may have been linked to the post-surgical follow-up, which could differ between hospitalization and external cares. For example, the consultation to assess strength recovery four months after surgery (question 1), in order to start running again, is not always proposed to all the patients in external care. Conversely, professional athletes have been excluded from the study because this strength assessment is usually performed earlier, at three months after surgery, in order to return to sport as soon as possible [22]. Fourthly, the patients of both groups have been operated on by six different surgeons and performed their rehabilitation with different physiotherapists. These health professionals may have provided different information to the patients, which may have caused an inequality of knowledge. Finally, we did not assess the clinical effect of our therapeutic education on clinical results, especially complications or joint evolution. It was due to the absence of comparability of the rehabilitation protocols between the two groups. Indeed, in case of clinical differences, the reasons could have been the therapeutic education, but also the type of rehabilitation. So, we chose to focus only on therapeutic education results, to avoid these misunderstandings.

## 5. Conclusions

Therapeutic patient education performed during hospitalization for rehabilitation enables patients to have a better knowledge of the stages from rehabilitation to return to sport and the risks of complication after anterior cruciate ligament reconstruction. Questionnaire administration and therapeutic education could have a positive impact on patients during the course of the rehabilitation to avoid psychological issues about their knee and the risks regarding return to sport. This could also lead to a better knowledge of the possible knee complications and allow earlier management if necessary. This early therapeutic approach seems important to manage the return to sports at risk. Therefore, further studies are necessary to know if this increase in knowledge allows a better return to sport or a reduction in the occurrence of complications after surgery. 

## Figures and Tables

**Table 1:** Self-report questionnaire and its correct answers in the original French version and English translation in italics. Patients had to give their degree of certitude concerning each answer directly in the last column: 100%: Tout à fait sûr (absolutely sure); 80%: Sûr (sure); 60%: Moyennement sûr (moderately sure); 50%: Pas sûr (not sure).

N°	Questions	Vrai/*True*	Faux/*False*	% Certitude
Q1	Je peux reprendre le footing à 4 mois avant évaluation musculaire *I can start jogging at 4 months, after muscle strength evaluation*		x	
Q2	Je peux continuer la pratique de la bicyclette si mon genou gonfle *I can continue cycling in case of knee swelling*		x	
Q3	Je peux reprendre un sport collectif à 6 mois avant évaluation musculaire *I can start a team sport at 6 months, before muscle strength evaluation*		x	
Q4	Je dois préserver les ischio-jambiers après 3 semaines post-opératoires *I have to protect my hamstrings 3 weeks after surgery*	x		
Q5	Je protège ma cicatrice pendant 1 an *I have to protect my scar for one year*	x		
Q6	La priorité de la rééducation en externat est la récupération totale des amplitudes articulaires de mon genou *The priority of the rehabilitation in external care is the recovery of the total knee range of motion*	x		
Q7	Je peux conduite une voiture si j’ai besoin des cannes pour marcher *I can drive a car if I need walking sticks*		x	
Q8	Il est habituel de reprendre le football à 4 mois post-opératoires *It is usual to restart soccer 4 months after surgery*		x	
Q9	Je débute la bicyclette à 2 mois si ma flexion de genou est complète *I can start cycling at 2 months if I have recovered knee flexion*	x		
Q10	La pratique de la natation est aussi efficace que le vélo pour renforcer les muscles *Swimming is as efficient as cycling for muscle strengthening*		x	
Q11	C’est le médecin du sport qui autorise la reprise de l’entrainement sportif *It is the sports physician who allows a return to training*	x		
Q12	Il existe toujours un risque de rompre mon nouveau ligament *There is always a risk of recurrence of ligament rupture*.	x		

**Table 2:** Patients’ characteristics.

Characteristics	Educated Group	Non Educated Group	*p*
Gender (F/M)	17/37	20/34	0.68 ^b^
Age (years)	26.7 ± 8.1	27.5 ± 8.1	0.49 ^a^
Weight (kg)	73.0 ± 13.0	72.0 ± 10.0	0.74 ^a^
Height (cm)	176.0 ± 7.0	177.0 ± 9.0	0.70 ^a^
Sports:			
- Soccer	26	18	
- Basketball	9	8	
- Handball	3	6	
- Ski	4	5	0.24 ^b^
- Volleyball	2	4	
- Combat sports	4	2	
- Other	6	11	
Operated side (R/L)	38/16	31/23	0.22 ^b^
Meniscus lesion	8	9	0.90 ^a^
Delay from sprain to surgery (months)	4.1 ± 5.0	5.0 ± 5.0	0.70 ^a^
Profession:			
- Craftsman	6	3	
- Manual worker	6	4	
- Clerk	6	5	
- Middle-grade manager	15	15	0.25 ^b^
- Upper-grade manager	7	11	
- Student	12	14	
- No job	2	2	

^a^ T-test; ^b^ χ^2^-test.

## Data Availability

The data presented in this study are available on request from the corresponding author. The data are not publicly available due to ethical reasons.
